# Mitochondrial Complex I deficiency: guilty in Parkinson’s disease

**DOI:** 10.1038/s41392-022-00983-3

**Published:** 2022-04-23

**Authors:** Melissa Vos

**Affiliations:** Institute of Neurogenetics, University of Luebeck, UKSH, Ratzeburger Allee 160, 23562 Luebeck, Germany

**Keywords:** Neurological disorders, Molecular neuroscience

Mitochondrial Complex I (MCI) deficiency has been abundantly observed in Parkinson’s disease (PD); however, it remained elusive if this was a consequence of the disease defects or rather disease-causing. Recent work in Nature by Gonzalez-Rodriguez and colleagues discovered MCI dysfunction to be sufficient to cause progressive parkinsonism.^1^

PD is characterized by the loss of dopaminergic neurons in the substantia nigra that is responsible for impaired motor function. Most PD patients are sporadic; yet, several genes have been identified that are causative of PD.^2^ Currently, PD is incurable and treatment strategies mainly consist of restoring dopamine levels in an attempt to provide symptomatic therapy. The lack of a cure or a treatment to stop the development originates from the lack of knowledge of the exact etiology of the pathogenesis. Notwithstanding, in the last few decades, immense research efforts have revealed several mechanisms that are affected in PD, including mitochondrial abnormalities.^3^

The first indication that dysfunctional mitochondria are involved in PD came from the observation that MPTP and rotenone, both blocking MCI function, developed irreversible parkinsonian-like symptoms. Furthermore, post mortem brains of PD patients displayed MCI defects. Interestingly, genes pathogenic variants in which cause recessive forms of PD encode proteins that are involved in mitochondrial function and mitochondrial quality control, further highlighting mitochondrial health to be an important regulator in PD. However, the question remained if mitochondrial abnormalities are a cause or consequence of the disease.

Recently, Gonzalez-Rodriguez and colleagues created mice with a conditional knockout in dopaminergic neurons for *ndufs2* (c*ndufs2*^*−/*−^), an essential subunit of complex I of the mitochondrial oxidative phosphorylation (OXPHOS), to uncover the role of MCI dysfunction in PD pathogenesis.^1^ In physiological conditions, c*ndufs2*^−*/*−^ did not induce alterations in the inner mitochondrial membrane (IMM) potential at postnatal day 20 (P20) through 40 (P40). However, the IMM completely collapsed following the blockade of ATP import via the adenine nucleotide transporter (ANT), suggesting a metabolic shift from OXPHOS to glycolysis. This was further supported by gene expression level experiments and cytosolic ATP/ADP ratio measurements using the genetically encoded sensor PercevalHR18. This metabolic shift did not induce loss of mitochondrial abundance, but rather led to an altered structure of mitochondrial cristae. These mitochondrial morphological alterations are, however, mild in comparison with the distorted mitochondrial morphology observed in other PD animal models, including loss of PINK1 and Parkin, in which mitochondria appear rounded and/or swollen.^4^

Remarkably, c*ndufs2*^*−/−*^ provoked downregulation of tyrosine hydroxylase (TH) protein levels in the dorsal striatum, an indication for decreased dopamine, but TH protein levels were unaffected in the SN and the ventral tegmental area (VTA) around P30. As a consequence, evoked striatal dopamine (DA) release was ablated, while no effect was detected in dendritic DA release in the SN. A progressive decline of TH positive cells was observed in the striatum and at P60, TH protein levels were decreased in the SN and VTA leading to reduced dendritic DA release. Nevertheless, the physiology of these neurons already displayed alterations at P30 that are manifested by the slowdown or stopping of autonomous pace-making recordings or the downregulation of pore-forming subunits of Cav1.3 channels that are responsible for Ca^2+^ transients to stimulate mitochondrial OXPHOS. Nonetheless, the physiological modification in the SN dopaminergic neurons was rather due to a downregulation and not a consequence of dopaminergic neuron loss, which is in contrast to what is observed in many animal models for PD in which DA rapidly declines. The detected alterations in c*ndufs2*^*-/-*^ did not result in large motor impairments at P30, but rather in minor difficulties in striatal learning and fine motor skills that were restored by levodopa treatment at P30; however, levodopa failed to induce a rescue of the striatal learning defects at P60. Furthermore, with aging, the mice developed increased abnormalities in locomotion that were only partially improved by levodopa treatment. Following these locomotion phenotypes, dopaminergic neurons appeared to be decreased in the SN. These data suggest that SN DA deficits are necessary to induce major locomotion difficulties, which is distinct from the generally accepted conception that striatal DA loss drives the locomotion problems. To further clarify this, the authors used a stereotaxic expression of aromatic-l-amino acid decarboxylase (AADC) that converts levodopa to DA in the SN or the striatum. Expression of AADC in the SN but also the striatum was able to improve locomotion following treatment of low doses of levodopa. Thus, depleted DA in both the striatum and the SN are necessary for the PD-like symptoms.

Current PD treatment exists mainly in the form of replacement therapy via e.g. levodopa administration; however, targeting the cause of PD would allow a halt of disease progression. The notion that a lack of proper MCI function is sufficient to induce progressive levodopa-responsive parkinsonism poses a novel therapeutic target in MCI. Previous studies using a genetic Pink1-related PD model in *drosophila melanogaster* revealed that stimulation of the OXPHOS using vitamin K2 or near-infrared light improves the displayed phenotypes^3^ and thus further supports the assumption that MCI-deficient forms of PD can be treated by stimulation of the OXPHOS. Several studies show other (parallel) pathways to play a role in PD, including a malfunctioning endo-lysosomal pathway as the seemingly primary defect. Altogether, these studies underscore the importance of understanding the exact underlying cause of the disease and the stratification of patients to specify the optimal treatment strategy.

**Fig. 1:**
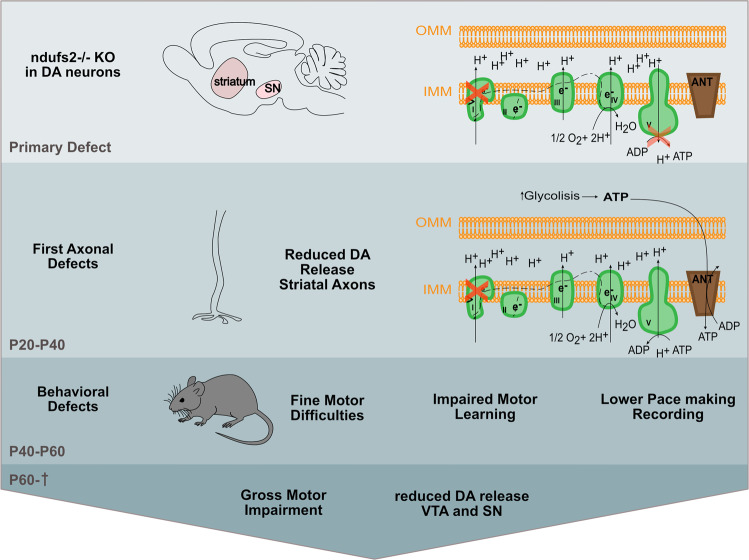
Schematic representation of the sequence of events following ndufs2^−/−^ knock out (KO) in dopaminergic neurons. Loss of ndufs2 in the dopaminergic neurons (Striatum and substantia nigra (SN) results initially in an inefficient OXPHOS (primary defect) followed by a metabolic shift to increased glycolysis and initial axonal defects presented as reduced release of dopamine in striatal axons (P20–P40). As a consequence, first behavioral phenotypes were observed in mice namely, they display fine motor abnormalities, impaired motor learning and lower pace making recordings (P40–P60). In a late stage (P60– death), also dopamine release from the SN and VTA is decreased further resulting into gross motor impairment

In summary, Gonzalez-Rodriguez and colleagues have created mice to generate MCI dysfunction that resulted in a metabolic shift demonstrating the plasticity capacity of DA neurons. Initially, it provoked a nigrostriatal dopaminergic effect that is accompanied by minor disabilities in motor learning and fine locomotion. These deficits progressively developed into an expanded loss of DA in the SN that, upon aging, led to levodopa-responsive locomotion difficulties (Fig. 1). Thus, these data provide compelling evidence that MCI deficiency is indeed sufficient to cause parkinsonism.
